# Energy Minimization for IRS-Assisted SWIPT-MEC System

**DOI:** 10.3390/s24175498

**Published:** 2024-08-24

**Authors:** Shuai Zhang, Yujun Zhu, Meng Mei, Xin He, Yong Xu

**Affiliations:** 1 School of Computer and Information, Anhui Normal University, Wuhu 241002, China; zup@ahnu.edu.cn (S.Z.); xin.he@ahnu.edu.cn (X.H.); yxull@ahnu.edu.cn (Y.X.); 2 School of Electronic and Information Engineering, Tongji University, Shanghai 200092, China; mei_meng@163.com

**Keywords:** IRS, MEC, SWIPT, energy consumption

## Abstract

With the rapid development of the internet of things (IoT) era, IoT devices may face limitations in battery capacity and computational capability. Simultaneous wireless information and power transfer (SWIPT) and mobile edge computing (MEC) have emerged as promising technologies to address these challenges. Due to wireless channel fading and susceptibility to obstacles, this paper introduces intelligent reflecting surfaces (IRS) to enhance the spectral and energy efficiency of wireless networks. We propose a system model for IRS-assisted uplink offloading computation, downlink offloading computation results, and simultaneous energy transfer. Considering constraints such as IRS phase shifts, latency, energy harvesting, and offloading transmit power, we jointly optimize the CPU frequency of IoT devices, offloading transmit power, local computation workload, power splitting (PS) ratio, and IRS phase shifts. This establishes a multi-variate coupled nonlinear problem aimed at minimizing IoT devices energy consumption. We design an effective alternating optimization (AO) iterative algorithm based on block coordinate descent, and utilize closed-form solutions, Dinkelbach-based Lagrange dual method, and semidefinite relaxation (SDR) method to minimize IoT devices energy consumption. Simulation results demonstrate that the proposed scheme achieves lower energy consumption compared to other resource allocation strategies.

## 1. Introduction

The internet of things (IoT) is a crucial component of future wireless networks, with a vast number of IoT devices connecting to the network in various ways. As technology rapidly advances, IoT has become one of the key technologies linking the world. However, within this extensive IoT ecosystem, many IoT devices face common challenges: limited battery capacity and computational power.

As one of the emerging technologies in IoT, mobile edge computing (MEC) enables IoT devices to offload their computational tasks to servers with adequate computing resources, potentially providing an effective solution to the aforementioned challenges. Deploying MEC at base station (BS) not only reduces task execution latency but also saves energy for IoT devices. Given that wireless channels degrade with distance and are susceptible to obstacles, this paper introduces intelligent reflecting surfaces (IRS) to enhance the spectral and energy efficiency of wireless networks.

Current research combining IRS and MEC has achieved notable results. Ref. [1] proposed a novel protocol that enabled the system to adaptively select the appropriate operating mode among energy harvesting mode, IRS auxiliary task offloading mode, and IRS standby task offloading mode based on the channel state information (CSI), IRS battery energy state, and user task queue state. This protocol formulated an optimization problem to minimize the energy consumption of user task offloading and computation. Ref. [2] investigated the problem of minimizing device latency in multi-device scenarios using the block coordinate descent method. Ref. [3] employed a partial offloading strategy and proposed a new performance evaluation metric for MEC systems, aiming to maximize total computation bits by jointly optimizing CPU frequency, offloading time allocation, devices transmit power, and IRS phase shifts. Ref. [4] introduced the first IRS-assisted binary offloading MEC system, addressing the problem of minimizing IoT devices energy consumption and proposing greedy and penalty-based algorithms while analyzing their applicable scenarios and characteristics. Ref. [5] tackled the issue of increased signaling overhead caused by device-specific IRS beamforming vectors, proposing a dynamic IRS beamforming framework to balance performance and signaling overhead, ultimately maximizing the total computation rate. Ref. [6] presented a weighted backhaul and power minimization problem, designing an IRS-assisted MEC system with caching by jointly optimizing the BS’s beamforming vector and IRS phase shifts vector. Ref. [7] introduced a new dual-IRS-assisted cooperative computing framework, where the source node offloads part of its computation tasks to multiple user devices through direct links, single-reflection links, and double-reflection links. Compared to traditional IRS-assisted MEC [8] used STAR-IRS, extending service coverage from half-space to full-space and addressing the problem of maximizing computation rates.

Due to the small size of most devices, their battery capacity is limited, posing challenges in terms of energy consumption. The aforementioned studies did not address the issues related to the limited battery capacity of IoT devices and the inconvenience of battery replacement. Consequently, some scholars have conducted the following research: Ref. [9] introduced wireless power transfer (WPT) technology, minimizing the total energy consumption of wireless devices by jointly optimizing WPT transmission time, IRS phase shifts for energy transfer, binary mode selection, CPU frequency, IRS phase shifts, as well as offloading time and power. Ref. [10] proposed a transmission strategy for IRS-assisted wireless powered sensor networks (WPSN). Sensor nodes first collected energy from an energy base station (BS) and then transmitted information using time division multiple access (TDMA). The strategy aimed to maximize total throughput under constraints on sensor node power, transmission time, and IRS discrete phase shifts. Ref. [11] presented a new energy optimization framework for IRS-assisted WP-MEC networks based on orthogonal frequency-division multiplexing (OFDM) and investigated the minimization of energy consumption in WP-MEC networks. Ref. [12] proposed a dynamic IRS beamforming framework to enhance the total throughput of IRS-assisted wireless powered communication networks (WPCN). Ref. [13] introduced an IRS-assisted wireless powered mobile edge computing and caching (WP-MECC) network, where the BS was equipped with local caches connected to the MEC server via backhaul links, allowing for data prefetching to facilitate edge computing capabilities.

The above studies focus on IRS-assisted system where terminals first perform downlink energy harvesting and then use the collected energy for uplink computational offloading, often neglecting the return of computation results. Simultaneous wireless information and power transfer (SWIPT) is considered an effective technology to address the issues of energy limitations and the inconvenience of battery replacement in wireless communication devices. SWIPT technology allows for the simultaneous wireless transmission of information and energy, enabling the return of computational offloading results along with energy transfer to IoT devices. This approach enhances energy utilization efficiency and extends the lifespan of IoT devices. Ref. [14] considered an IRS and SWIPT-assisted NOMA-MEC network. By jointly optimizing the IRS passive beamforming vector, power splitting (PS) ratio, relay power allocation factor, offloading transmit power, and offloading task volume, the study aimed to minimize network task computation latency and energy consumption. Ref. [15] simultaneously considered energy harvesting and task offloading. Under quality of service constraints, it jointly optimized IRS reflection coefficients and task allocation strategies to maximize user energy savings.

In summary, few scholars have explored the integration of IRS, SWIPT, and MEC technologies into a unified system architecture. Additionally, there is limited research on minimizing IoT devices energy consumption using an IRS-assisted SWIPT-MEC system with a PS architecture for IoT devices. To address the limitations in computational capacity of IoT devices while extending network lifespan, this paper delves into such a system. It considers the return of MEC-processed results to IoT devices via SWIPT technology and proposes a resource allocation scheme to minimize IoT devices energy consumption.

The main research contributions of this paper are as follows:

1. System model development: We propose a system model for IRS-assisted uplink offloading computation, downlink offloading computation results, and simultaneous energy transfer.Under the constraints of IRS phase shifts, latency, energy harvesting, and offloading transmit power, we jointly optimize the IoT devices’ CPU frequency, offloading transmit power, local computation workload, PS ratio, and IRS phase shifts. This constructs a multi-variable coupled nonlinear problem [16,17,18,19,20] aimed at minimizing IoT devices energy consumption.
2. Algorithm design: To solve this problem, we design an effective block coordinate descent-based alternating optimization (AO) iterative algorithm. Specifically, we decompose the multi-variable coupled nonlinear problem into two subproblems and sequentially optimize the variables in these subproblems using the AO algorithm. First, we use closed-form solutions to solve the optimal CPU frequency for IoT devices, employ the convex optimization tool CVX to solve the optimal local computation workload allocation problem, and use Dinkelbach-based Lagrange dual method to solve for the optimal offloading transmit power of IoT devices. Next, we optimize the PS ratio using closed-form solutions and optimize the IRS phase shifts using the semidefinite relaxation (SDR) method.
3. Simulation results: The simulation results demonstrate that the IRS can effectively reduce the energy consumption of IoT devices in the SWIPT-MEC system. Compared to other benchmark schemes, the proposed scheme exhibits superior performance.

Notations: Boldface lowercase letters denote vectors and uppercase letters are matrices. $C^{M\times N}$ represents a complex matrix with the dimensional of M×N. ${\left(.\right)}^{H}$ denote the conjugate transpose operations Tr(.) stands for trace of a matrix. diag(.) denotes a vector that consists of the diagonal elements of a matrix or a diagonal matrix where the diagonal elements are from a vector. I and 0 denote the identity matrix and zero matrix. C⪰0 indicates that C is a positive semidefinite matrix. a∼CN(0,1) denotes that s follows the complex Gaussian distribution with zero-mean and unit variance.

## 2. System Model

**Figure 1:**
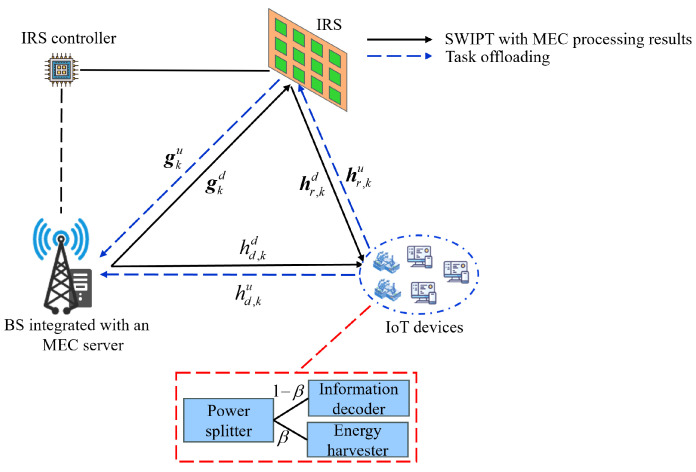
IRS-assisted SWIPT-MEC system.

The system model in this paper consists of a BS with a single antenna equipped with an MEC server, an IRS with *N* reflecting elements, and *K* IoT devices with PS architecture, each equipped with a single antenna. In this architecture, the received signal power at IoT devices is split into components: β(0≤β≤1) is used for energy harvesting, and 1−β is used for information decoding. The system model is illustrated in Figure 1.

**Figure 2:**
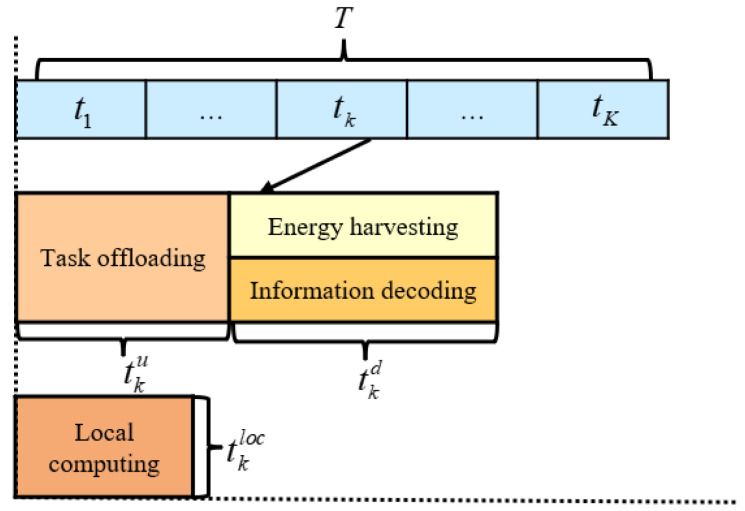
The *k*-th IoT device working time slot.

Assuming each IoT device starts with a fully charged battery, during each coherent interval, IoT devices operate in a first-in-first-out (FIFO) mode. They first perform uplink computational offloading while simultaneously conducting local computations. Subsequently, the BS transmits energy downlink to the IoT devices along with the computational results from the MEC server. The operational time slot for the *k*-th IoT device is illustrated in Figure 2. Local computation tasks are denoted as $L_{k}^{u}$ during local computation slot $t_{k}^{loc}$. Each IoT device k∈{1,,K} unloads computation tasks $\left(L_{k}-L_{k}^{u}\right)\left(0\leq L_{k}^{u}\leq L_{k}\right)$ to the MEC server carried by the BS within duration $t_{k}^{u}$, where *K* is the total number of IoT devices and $L_{k}$ is the total computation task for the *k*-th IoT device. Next, within time $t_{k}^{d}$, the BS transmits completed computation tasks $L_{k}^{r}$ from the MEC server to the IoT devices and carries energy information $L_{k}^{r}=\alpha_{k}\left(L_{k}-L_{k}^{u}\right)$, $\alpha_{k}\left(0\leq \alpha_{k}\leq 1\right)$ as a proportion of the return task assignment. Due to the powerful computing power of the MEC server, ignore its computational offload task time. Due to the limited computing power of the IoT devices itself, it is assumed that the local computation latency is less than the offloading latency, i.e., $0\leq t_{k}^{loc}\leq t_{k}^{u}$.

This paper assumes channel reciprocity and quasi-static channel conditions, meaning that the CSI for both the uplink and downlink remains unchanged and identical within the considered time period. Additionally, CSI is assumed to be fully known. For the uplink task offloading, the diagonal phase shifts matrix of the *k*-th time slot for the IRS is denoted as $\Theta_{k}^{d}=\mathrm{diag}\left(e^{j\theta_{k,1}^{d}},e^{j\theta_{k,2}^{d}},\ldots ,e^{j\theta_{k,N}^{d}}\right)$, where j represents the imaginary unit, $\theta_{k,n}^{d}\in \left[\begin{array}{c} 0,2\pi \end{array}\right],n\in \left\{\begin{array}{c} 1,\ldots ,N \end{array}\right\}$ is the phase shifts of the *n*-th reflecting element. Let $\theta_{k}^{d}=\left(e^{j\theta_{k,1}^{d}},e^{j\theta_{k,2}^{d}},\ldots ,e^{j\theta_{k,N}^{d}}\right)$. Similar to the uplink task offloading, for the simultaneous downlink energy and computation offloading task results transmission, the diagonal phase shifts matrix of the *k*-th time slot for the IRS is denoted as $\Theta_{k}^{u}=diag\left(e^{j\theta_{k,1}^{u}},e^{j\theta_{k,2}^{u}},\ldots ,e^{j\theta_{k,N}^{u}}\right)$, where $\theta_{k,n}^{u}\in \left[0,2\pi \right],n\in \left\{1,\ldots ,N\right\}$ is the phase shifts of the *n*-th reflecting element. Let $\theta_{k}^{u}=\left(e^{j\theta_{k,1}^{u}},e^{j\theta_{k,2}^{u}},\ldots ,e^{j\theta_{k,N}^{u}}\right)$. For uplink communication, $g_{k}^{u}\in C^{N\times 1}$ represents the channel from the IRS to the BS. $h_{r,k}^{u}\in C^{1\times N}$ represents the channel from the *k*-th IoT device to the IRS. $h_{d,k}^{u}\in C^{1\times 1}$ represents the channel from the *k*-th IoT device to the BS. Similarly, for downlink communication, $g_{k}^{d}\in C^{l\times N}$ represents the channel from the BS to the IRS. $h_{r,k}^{d}\in C^{N\times 1}$ represents the channel from the IRS to the *k*-th IoT device. $h_{d,k}^{d}\in C^{1\times 1}$ represents the channel from the BS to the *k*-th IoT device. $s_{k}^{u}$ represents the signal from the *k*-th IoT device to the BS, satisfying $|s_{k}^{u}{|}^{2}=1$. Similarly, $s_{k}^{d}$ represents the signal from the BS to the *k*-th IoT device, satisfying $|s_{k}^{d}{|}^{2}=1$. Additionally, $p_{k}^{u}$ and $p_{k}^{d}$ respectively represent the transmit power of the *k*-th IoT device and the BS. Furthermore, the BS’s additive white Gaussian noise (AWGN) is represented by $n_{bs}$, with covariance matrix $\delta_{bs}^{2}I_{M}$. Similarly, the AWGN for the *k*-th IoT device and the PS receiver are represented respectively by $n_{k}^{d}$ and $n_{ps}$, with covariances $\delta_{k}^{2}$ and $\delta_{ps}^{2}$. Finally, *B* represents the system bandwidth.

### 2.1. Uplink Communication

In the uplink communication process, IoT devices sequentially offload computational tasks to the MEC server carried by the BS. Therefore, the *k*-th IoT device transmits a signal $s_{k}^{u}$ to the BS within time interval $t_{k}^{u}$ using offloading transmit power $p_{k}^{u}$, after which the BS transmits the signal $s_{k}^{d}$ back to the *k*-th IoT device at transmit power $p_{k}^{d}$. It is assumed that self-interference at the BS is mitigated using self-interference cancellation (SIC) technology. Thus, the signal received at the BS can be represented as

(1) $$y_{k}^{u}=\sqrt{p_{k}^{u}}\left(h_{d,k}^{u}+h_{r,k}^{u}\Theta_{k}^{u}g_{k}^{u}\right)s_{k}^{u}+n_{bs}.$$

The received signal power is expressed as

(2) $$P_{k}^{u}=p_{k}^{u}|h_{d,k}^{u}+h_{r,k}^{u}\Theta_{k}^{u}g_{k}^{u}{|}^{2}+\delta_{bs}^{2}.$$

So, the uplink offloading rate can be expressed as

(3) $$R_{k}^{u}=B\log_{2}\left(1+\frac{p_{k}^{u}|h_{d,k}^{u}+h_{r,k}^{u}\Theta_{k}^{u}g_{k}^{u}{|}^{2}}{\delta_{bs}^{2}}\right).$$

The offloading computation time of the *k*-th IoT device can be represented as

(4) $$t_{k}^{u}=\frac{L_{k}-L_{k}^{u}}{R_{k}^{u}}.$$

The energy consumption of the *k*-th IoT device during offloading computation can be represented as

(5) $$E_{k}^{u}=p_{k}^{u}\frac{L_{k}-L_{k}^{u}}{R_{k}^{u}}.$$

Assuming IoT devices employ half-duplex (HD) technology, hence, uplink offloading computation and downlink reception of energy and information cannot occur simultaneously. This paper uniformly divides the coherence time *T* of the channels among *K* IoT devices, ensuring that the transmission times of uplink and downlink channels are less than the allocated time slot for each IoT device, i.e.,

(6) $$0\leq t_{k}^{u}+t_{k}^{d}\leq \frac{T}{K}.$$

This paper focuses on exploring the energy consumption of IoT devices. Given that the BS’s downlink transmission rate significantly exceeds the uplink offloading rate of IoT devices, we assume that the time for MEC to coordinate the transmission of processing results and energy back to IoT devices is negligible. Therefore, the time slot constraint for IoT devices can be expressed as

(7) $$0\leq t_{k}^{u}\leq \frac{T}{K}.$$

The local computation time of the *k*-th IoT device, where $C_{k}$ represents the number of CPU cycles required per bit for computation, is expressed as

(8) $$t_{k}^{loc}=\frac{C_{k}L_{k}^{u}}{f_{k}}.$$

The local energy consumption of the *k*-th IoT device can be expressed as

(9) $$E_{k}^{loc}=\kappa_{k}C_{k}L_{k}^{u}{\left(f_{k}\right)}^{2}.$$

where $\kappa_{k}$ represents the effective capacitance coefficient of the processor chip in the *k*-th IoT device, which depends on the chip’s architecture.

If IoT devices have sufficient energy to offload computation tasks and to perform local computation tasks by harvesting energy from the BS, then the energy consumption during both computation offloading and local computation by IoT devices should meet the following condition

(10) $$E_{k}^{u}+E_{k}^{loc}\leq E_{k}^{h}.$$

### 2.2. Downlink Communication

In the downlink communication process, the received signal at the *k*-th IoT device comprises two components: the radio frequency signal from the BS and the AWGN at the IoT devices. This process is similar to the uplink communication.

The signal received by the *k*-th IoT device can be expressed as

(11) $$y_{k}^{d}=\sqrt{p_{k}^{d}}\left(h_{d,k}^{d}+g_{k}^{d}\Theta_{k}^{d}h_{r,k}^{d}\right)s_{k}^{d}+n_{k}^{d}.$$

The received signal power at the *k*-th IoT device can be derived as follows

(12) $$P_{k}^{d}=p_{k}^{d}|h_{d,k}^{d}+g_{k}^{d}\Theta_{k}^{d}h_{r,k}^{d}{|}^{2}+\delta_{k}^{2}.$$

The information decoding signal of the *k*-th IoT device can be expressed as

(13) $$y_{k}^{ID}=\left(1-\beta_{k}\right)\left(\sqrt{p_{k}^{d}}\left(h_{d,k}^{d}+g_{k}^{d}\Theta_{k}^{d}h_{r,k}^{d}\right)s_{k}^{d}+n_{k}^{d}\right)+z_{k},$$

where $z_{k}∼\mathrm{CN}\left(0,\delta_{k}^{2}\right)$ is the noise generated by the decoding of the signal.

The downlink transmission rate can be expressed as

(14) $$R_{k}^{d}=B\log_{2}\left(1+\frac{\left(1-\beta_{k}\right)p_{k}^{d}|h_{d,k}^{d}+g_{k}^{d}\Theta_{k}^{d}h_{r,k}^{d}{|}^{2}}{\delta_{k}^{2}}\right).$$

The signal received at the energy harvesting receiver can be expressed as

(15) $$y_{k}^{EH}=\beta_{k}\left(\sqrt{p_{k}^{d}}{\left(h_{d,k}^{d}+g_{k}^{d}\Theta_{k}^{d}h_{r,k}^{d}\right)}^{d}s_{k}^{d}+n_{k}^{d}\right).$$

According to the materials used, the energy harvesting model can be considered as either linear or nonlinear. Rechargeable batteries can be modeled using an ideal linear model, while devices based on capacitors employ nonlinear modeling. This paper assumes IoT devices are equipped with rechargeable batteries; hence, the energy harvesting model is linear. Furthermore, since the noise power is much smaller than the received signal power, this paper does not consider energy harvested from noise power. Thus, the energy harvested by the *k*-th IoT device is represented as

(16) $$E_{k}^{h}=\eta_{k}\beta_{k}p_{k}^{d}|h_{d,k}^{d}+g_{k}^{d}\Theta_{k}^{d}h_{r,k}^{d}{|}^{2},$$

where $\eta_{k}\in \left(0,1\right)$ is the energy conversion efficiency factor of the *k*-th IoT device.

## 3. Problem Modeling

In this paper, the objective is to minimize the total energy consumption of devices by jointly optimizing the PS ratio, the local computation workload, the IRS phase shifts matrix, the offloading transmit power of IoT devices, and the CPU frequency. Since all IoT devices are independent of each other in each time slot, this paper analyzes the energy consumption of the *k*-th IoT device only. That is

(17) $$\begin{array}{cc} \; & \min_{f_{k},L_{k}^{u},p_{k}^{u},\beta_{k},\theta_{k}^{d},\theta_{k}^{u}}E_{k}^{u}+E_{k}^{loc}\\ s.t. & \;C_{1}:E_{k}^{u}+E_{k}^{loc}\leq E_{k}^{h},\\ \; & \;C_{2}:0\leq f_{k}\leq f_{max},\\ \; & \;C_{3}:0\leq \beta_{k}\leq 1,\\ \; & \;C_{4}:0\leq L_{k}^{u}\leq L_{k},\\ \; & \;C_{5}:p_{\min}^{u}\leq p_{k}^{u}\leq p_{\max}^{u},\\ \; & \;C_{6}:0\leq t_{k}^{loc}\leq t_{k}^{u},\\ \; & \;C_{7}:0\leq t_{k}^{u}\leq \frac{T}{K},\\ \; & \;C_{8}:|\theta_{k,n}^{d}|=1,\\ \; & \;C_{9}:|\theta_{k,n}^{u}|=1. \end{array}$$

In this context, $f_{max}$ represents the maximum CPU frequency of the IoT device, while $p_{min}^{u}$ and $p_{max}^{u}$ denote the minimum and maximum transmit power for offloading, respectively. Constraints $C_{1}$ indicates that the local energy consumption and the offloaded computational energy consumption of the *k*-th IoT device are subject to the energy harvesting constraints; Constraints $C_{2}$ indicates the limiting constraints of the CPU frequency; Constraints $C_{3}$ indicates the PS ratio constraints; Constraints $C_{4}$ indicates the local computational workload constraints; Constraints $C_{5}$ indicates the limiting constraints of the offloaded transmit power; Constraints $C_{6}$ indicates that the local computational latency is less than the offloaded computational latency constraints; Constraints $C_{7}$ indicates that the IoT device offload computation latency is less than the time slot constraint assigned to the *k*-th IoT device; Constraints $C_{8}$ and $C_{9}$ indicates the phase shifts constraints for each reflection unit of the IRS.

## 4. Problem Optimization

**Figure 3:**
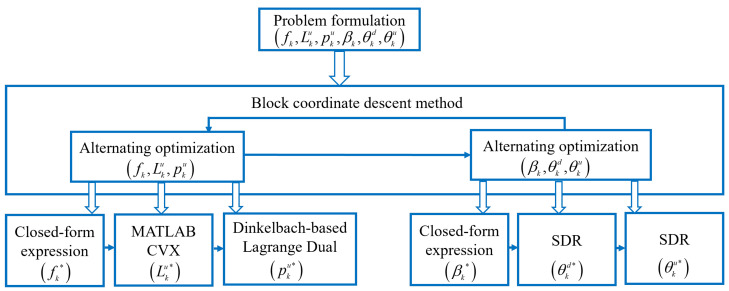
The flow chart of the proposed optimization method.

Due to the non-convex nature of Equation (17), which involves coupled variables, direct solution is not feasible. In this section, we employ an iterative algorithm based on block coordinate descent for AO, decomposing Equation (18) into two sub-problems. Specifically, first, given the IRS phase shifts and the PS ratio, we solve for the CPU frequency of IoT devices, local computation workload, and offloading transmit power using the AO algorithm. Next, based on the obtained CPU frequency, local computation workload, and offloading transmit power of IoT devices, we solve for IRS phase shifts and PS ratio using the AO algorithm. The corresponding flow chart is illustrated in Figure 3.

### 4.1. Optimization of CPU Frequency, Local Computation Workload, and Offloading Transmit Power of IoT Devices

Under fixed IRS phase shifts and PS ratio, the offloading transmit power, local computation workload, and CPU frequency of IoT devices are optimized. It is evident from $f_{k}=\frac{C_{k}L_{k}^{u}}{t_{k}^{loc}}$ that when the uplink offloading time equals the local computation time, the local energy consumption of the *k*-th IoT device is minimized, denoted as $t_{k}^{loc}=t_{k}^{u}=\frac{T}{K}$. The optimal CPU frequency is represented as $f_{k}^{*}=\frac{KC_{k}L_{k}^{u}}{T}$, and the local energy consumption of IoT devices is denoted as $E_{k}^{loc}=\frac{\kappa_{k}K^{2}C_{k}^{3}{\left(L_{k}^{u}\right)}^{3}}{T^{2}}$. Thus, the problem is described as

(18) $$\begin{array}{cc} \; & \min_{L_{k}^{u},p_{k}^{u}}\;p_{k}^{u}\frac{\left(L_{k}-L_{k}^{u}\right)}{R_{k}^{u}}+\frac{\kappa_{k}K^{2}C_{k}^{3}{\left(L_{k}^{u}\right)}^{3}}{T^{2}}\\ s.t. & \;C_{4},C_{5},\\ \; & \;C_{1}:p_{k}^{u}\frac{\left(L_{k}-L_{k}^{u}\right)}{R_{k}^{u}}+\frac{\kappa_{k}K^{2}C_{k}^{3}{\left(L_{k}^{u}\right)}^{3}}{T^{2}}\leq \eta_{k}\beta_{k}p_{k}^{d}|h_{d,k}^{d}+g_{k}^{d}\Theta_{k}^{d}h_{r,k}^{d}{|}^{2}. \end{array}$$

Equation (18) above remains a non-convex problem with multi-variable coupling, making direct solution challenging. By fixing variable $p_{k}^{u}$ and optimizing variable $L_{k}^{u}$, it can be reformulated as follows

(19) $$\begin{array}{cc} \; & \min_{L_{k}^{u}}\;p_{k}^{u}\frac{\left(L_{k}-L_{k}^{u}\right)}{R_{k}^{u}}+\frac{\kappa_{k}K^{2}C_{k}^{3}{\left(L_{k}^{u}\right)}^{3}}{T^{2}}\\ s.t. & \;C_{1},C_{4}. \end{array}$$

In this case, the problem involves a combination of two convex functions, thus it remains convex and can be solved using the MATLAB CVX (Version 2.2) tool.

Subsequently, fixing variable $L_{k}^{u}$ and optimizing variable $p_{k}^{u}$, with $p_{k}^{u}b_{k}=\frac{p_{k}^{u}|h_{d,k}^{u}+h_{r,k}^{u}\Theta_{k}^{u}g_{k}^{u}{|}^{2}}{\delta_{bs}^{2}}$, Equation (18) transforms into

(20) $$\begin{array}{cc} \; & \min_{P_{k}^{u}}\;p_{k}^{u}\frac{\left(L_{k}-L_{k}^{u}\right)}{R_{k}^{u}}\\ s.t. & \;C_{5},\\ \; & \;C_{1}:p_{k}^{u}\frac{\left(L_{k}-L_{k}^{u}\right)}{B\log_{2}\left(1+p_{k}^{u}b_{k}\right)}\leq \eta_{k}\beta_{k}p_{k}^{d}|h_{d,k}^{d}+g_{k}^{d}\Theta_{k}^{d}h_{r,k}^{d}{|}^{2}-\frac{\kappa_{k}K^{2}C_{k}^{3}{\left(L_{k}^{u}\right)}^{3}}{T^{2}}. \end{array}$$

Due to the non-convex nature of the aforementioned problem, this paper employs the Dinkelbach method to alter the objective function’s fractional structure, introducing a relaxation variable λ, thereby restructuring Equation (20) as follows

(21) $$\begin{array}{cc} \; & \min_{p_{k}^{u}}\;\frac{L_{k}-L_{k}^{u}}{B}\left(p_{k}^{u}\lambda -\log_{2}\left(1+p_{k}^{u}b_{k}\right)\right)\\ s.t. & \;C_{5},\\ \; & \;C_{1}:\frac{L_{k}-L_{k}^{u}}{B}\left(p_{k}^{u}\lambda -\log_{2}\left(1+p_{k}^{u}b_{k}\right)\right)\leq \eta_{k}\beta_{k}p_{k}^{d}|h_{d,k}^{d}+g_{k}^{d}\Theta_{k}^{d}h_{r,k}^{d}{|}^{2}-\frac{\kappa_{k}K^{2}C_{k}^{3}{\left(L_{k}^{u}\right)}^{3}}{T^{2}}, \end{array}$$

where $\lambda =\frac{\log_{2}\left(1+p_{k}^{u}b_{k}\right)}{p_{k}^{u}}$, $F\left(p_{k}^{u},\lambda \right)=\frac{L_{k}-L_{k}^{u}}{B}\left(p_{k}^{u}\lambda -\log_{2}\left(1+p_{k}^{u}b_{k}\right)\right)$, and optimizing Equation (21) leads to the Lagrange function

(22) $$L\left(p_{k}^{u},\lambda ,\varepsilon \right)=F\left(p_{k}^{u},\lambda \right)+\varepsilon \left(F\left(p_{k}^{u},\lambda \right)+\frac{\kappa_{k}K^{2}C_{k}^{3}{\left(L_{k}^{u}\right)}^{3}}{T^{2}}-\eta_{k}\beta_{k}p_{k}^{d}|h_{d,k}^{d}+g_{k}^{d}\Theta_{k}^{d}h_{r,k}^{d}{|}^{2}\right),$$

Among these, ε acts as a Lagrange multiplier, and $p_{k}^{u}\in \left[p_{min}^{u},p_{max}^{u}\right]$. Transform Equation (20) into an optimization problem with Lagrange multipliers, specifically solving

(23) $$\begin{array}{cc} \min_{p_{k}^{u},\lambda }\max_{\varepsilon }L\left(p_{k}^{u},\lambda ,\varepsilon \right) & =\min_{p_{k}^{u},\lambda }\max_{\varepsilon }F\left(p_{k}^{u},\lambda \right)\\ \; & \;+\varepsilon \left(F\left(p_{k}^{u},\lambda \right)+\frac{\kappa_{k}K^{2}C_{k}^{3}{\left(L_{k}^{u}\right)}^{3}}{T^{2}}-\eta_{k}\beta_{k}p_{k}^{d}{\left|h_{d,k}^{d}+g_{k}^{d}\Theta_{k}^{d}h_{r,k}^{d}\right|}^{2}\right). \end{array}$$

By fixing variable λ, the optimal solution $p_{k}^{u^{*}}$ can be obtained through $\frac{\partial L(p_{k}^{u},\lambda ,\varepsilon )}{\partial p_{k}^{u}}=0$, represented as

(24) $$p_{k}^{u^{*}}={\left[\frac{1}{\left(ln2\right)\lambda }-\frac{1}{b_{k}}\right]}_{p_{min}^{u}}^{p_{max}^{u}},$$

where ${\left[x\right]}_{a}^{b}=\max\left\{\min\left\{x,b\right\},a\right\}$. The specific algorithmic procedure is depicted in Algorithm 1.
**Algorithm 1: Dinkelbach-Based Lagrange Dual Method**Initialize iteration count t=0, $p_{k}^{u}\left(t\right)$, $b_{k}\left(t\right)$ and set the stopping criterion for iterations.**Loop**   Initialize $\lambda \left(t\right)=\frac{\log_{2}\left(1+p_{k}^{u}\left(t\right)b_{k}\left(t\right)\right)}{p_{k}^{u}\left(t\right)}$;   Solve Equation (21) using the Lagrange dual method;   Initialize Lagrange multipliers ε(t);   **Loop**      Solve for $p_{k}^{u}\left(t\right)$ and update $p_{k}^{u^{*}}\left(t+1\right)={\left[\frac{1}{\left(ln2\right)\lambda \left(t\right)}-\frac{1}{b_{k}\left(t\right)}\right]}_{p_{min}^{u}}^{p_{max}^{u}}$;      Update Lagrange multipliers ε(t+1);      Until the Lagrange dual function converges.   **End**   Update $\lambda \left(t+1\right)=\frac{\log_{2}\left(1+p_{k}^{u}\left(t+1\right)b_{k}\left(t+1\right)\right)}{p_{k}^{u}\left(t+1\right)}$;**Until** the Dinkelbach optimality condition is satisfied.

### 4.2. Optimization of IRS Phase Shifts and PS Ratio

By fixing the offloading transmit power of IoT devices, local computation workload, and CPU frequency, optimize the IRS phase shifts and PS ratio. Thus, the optimization problem is described as

(25) $$\begin{array}{cc} \; & \min_{\beta_{k},\theta_{k}^{d},\theta_{k}^{u}}\;p_{k}^{u}\frac{L_{k}-L_{k}^{u}}{B\log_{2}\left(1+\frac{p_{k}^{u}|h_{d,k}^{u}+h_{r,k}^{u}\Theta_{k}^{u}g_{k}^{u}{|}^{2}}{\delta_{bs}^{2}}\right)}\\ s.t. & \;C_{3},C_{8},C_{9},\\ \; & \;C_{1}:p_{k}^{u}\frac{L_{k}-L_{k}^{u}}{B\log_{2}\left(1+\frac{p_{k}^{u}|h_{d,k}^{u}+h_{r,k}^{u}\Theta_{k}^{u}g_{k}^{u}{|}^{2}}{\delta_{bs}^{2}}\right)}+\frac{\kappa_{k}K^{2}C_{k}^{3}{\left(L_{k}^{u}\right)}^{3}}{T^{2}}\leq \eta_{k}\beta_{k}p_{k}^{d}|h_{d,k}^{d}+g_{k}^{d}\Theta_{k}^{d}h_{r,k}^{d}{|}^{2}. \end{array}$$

At this point, Equation (25) remains a complex non-convex problem. Therefore, fixing variable $\theta_{k}^{u}$ and $\theta_{k}^{d}$, optimizing variable $\beta_{k}$, transforms the problem into an optimization problem where the objective function does not include decision variables, Equation (25) transforms into

(26) $$\begin{array}{cc} \; & Find\;\beta_{k}\\ s.t. & \;C_{3},\\ \; & \;C_{1}:\frac{E_{k}^{u}+E_{k}^{loc}}{\eta_{k}\beta_{k}p_{k}^{d}|h_{d,k}^{d}+g_{k}^{d}\Theta_{k}^{d}h_{r,k}^{d}{|}^{2}}\leq \beta_{k}. \end{array}$$

Due to $\frac{E_{k}^{u}+E_{k}^{loc}}{\eta_{k}\beta_{k}p_{k}^{d}|h_{d,k}^{d}+g_{k}^{d}\Theta_{k}^{d}h_{r,k}^{d}{|}^{2}}>0$, it is evident that when $\beta_{k}^{*}=\min\left\{\frac{E_{k}^{u}+E_{k}^{loc}}{\eta_{k}\beta_{k}p_{k}^{d}|h_{d,k}^{d}+g_{k}^{d}\Theta_{k}^{d}h_{r,k}^{d}{|}^{2}},1\right\}$ is minimized variable $\beta_{k}$, the energy consumption of IoT devices is minimized.

Next, with variable $\beta_{k}$ fixed and variable $\theta_{k}^{u}$ and $\theta_{k}^{d}$ optimized, Equation (25) can be transformed into

(27) $$\begin{array}{cc} \; & \min_{\theta_{k}^{d},\theta_{k}^{u}}\;p_{k}^{u}\frac{L_{k}-L_{k}^{u}}{B\log_{2}\left(1+\frac{p_{k}^{u}{\left|h_{d,k}^{u}+h_{r,k}^{u}\Theta_{k}^{u}g_{k}^{u}\right|}^{2}}{\delta_{bs}^{2}}\right)}\\ s.t. & \;C_{8},C_{9},\\ \; & \;C_{1}:p_{k}^{u}\frac{L_{k}-L_{k}^{u}}{B\log_{2}\left(1+\frac{p_{k}^{u}{\left|h_{d,k}^{u}+h_{r,k}^{u}\Theta_{k}^{u}g_{k}^{u}\right|}^{2}}{\delta_{bs}^{2}}\right)}+\frac{\kappa_{k}K^{2}C_{k}^{3}{\left(L_{k}^{u}\right)}^{3}}{T^{2}}\leq \eta_{k}\beta_{k}p_{k}^{d}|h_{d,k}^{d}+g_{k}^{d}\Theta_{k}^{d}h_{r,k}^{d}{|}^{2}. \end{array}$$

Given that $\theta_{k}^{u}$ and $\theta_{k}^{d}$ is mutually coupled, we first fix $\theta_{k}^{u}$. Optimizing the downlink IRS phase shifts $\theta_{k}^{d}$ transforms Equation (27) to

(28) $$\begin{array}{cc} \; & Find\;\theta_{k}^{d}\\ s.t. & \;C_{8},\\ \; & \;C_{1}:E_{k}^{u}+E_{k}^{loc}\leq \eta_{k}\beta_{k}p_{k}^{d}|h_{d,k}^{d}+g_{k}^{d}\Theta_{k}^{d}h_{r,k}^{d}{|}^{2}. \end{array}$$

Let $\hat{\theta_{k}^{d}}=\left[\theta_{k}^{d},1\right]$, $h_{k}^{d}={\left[diag\left(g_{k}^{d}\right)h_{r,k}^{d},h_{d,k}^{d}\right]}^{H}$, then $|h_{d,k}^{d}+g_{k}^{d}\Theta_{k}^{d}h_{r,k}^{d}|=h_{k}^{d}\hat{\theta_{k}^{d}}$, Equation (28) can then be rewritten as

(29) $$\begin{array}{cc} \; & Find\;\hat{\theta_{k}^{d}}\\ s.t. & \;C_{1}:E_{k}^{u}+E_{k}^{loc}\leq \eta_{k}\beta_{k}p_{k}^{d}|h_{k}^{d}\hat{\theta_{k}^{d}}{|}^{2},\\ \; & \;C_{8}:{\left[{\hat{\theta_{k}^{d}}}^{H}\hat{\theta_{k}^{d}}\right]}_{mn}=1,\forall n\in N. \end{array}$$

To address the nonlinear equality constraint problem in Equation (29), introduce variable $\hat{\Theta_{k}^{d}}={\hat{\theta_{k}^{d}}}^{H}\hat{\theta_{k}^{d}}$, $\hat{H_{k}^{d}}=h_{k}^{d}h_{k}^{d^{H}}$, thereby transforming the problem into

(30) $$\begin{array}{cc} \; & Find\;\hat{\Theta_{k}^{d}}\\ s.t. & \;C_{1}:E_{k}^{u}+E_{k}^{loc}\leq \eta_{k}\beta_{k}p_{k}^{d}Tr\left(\hat{H_{k}^{d}}\hat{\Theta_{k}^{d}}\right),\\ \; & \;C_{8}:{\left[\hat{\Theta_{k}^{d}}\right]}_{nn}=1,\forall n\in N,\\ \; & \;C_{10}:\hat{\Theta_{k}^{d}}⪰0,\\ \; & \;C_{11}:Rank\left(\hat{\Theta_{k}^{d}}\right)=1. \end{array}$$

Since $C_{11}$ imposes a non-convex rank-1 constraint, its restriction can be relaxed using the SDR algorithm, thus ultimately converting it into a convex optimization problem solvable with the CVX tool. However, the optimal solution’s rank being 1 cannot be guaranteed, hence further Gaussian randomization is employed to restore a rank-1 solution.

Subsequently, with $\theta_{k}^{d}$ fixed, optimize the uplink IRS phase shifts $\theta_{k}^{u}$. As the task offloading rate of the *k*-th IoT device increases with the phase shifts, the problem can be simplified to

(31) $$\begin{array}{cc} \; & \max_{\theta_{k}^{u}}\;|h_{d,k}^{u}+h_{r,k}^{u}\Theta_{k}^{u}g_{k}^{u}{|}^{2}\\ s.t. & \;C_{9},\\ \; & \;C_{1}:p_{k}^{u}\frac{L_{k}-L_{k}^{u}}{B\log_{2}\left(1+\frac{p_{k}^{u}|h_{d,k}^{u}+h_{r,k}^{u}\Theta_{k}^{u}g_{k}^{u}{|}^{2}}{\delta_{bs}^{2}}\right)}+E_{k}^{loc}\leq E_{k}^{h}. \end{array}$$

Let $\hat{\theta_{k}^{u}}=\left[\theta_{k}^{u},1\right]$, $h_{k}^{u}={\left[diag\left(g_{k}^{u}\right)h_{r,k}^{u},h_{d,k}^{u}\right]}^{H}$, then $|h_{d,k}^{u}+g_{k}^{u}\Theta_{k}^{u}h_{r,k}^{u}|=h_{k}^{u}\hat{\theta_{k}^{u}}$, Equation (31) can then be rewritten as

(32) $$\begin{array}{cc} \; & \max_{\hat{\theta_{k}^{u}}}\;|h_{k}^{u}\hat{\theta_{k}^{u}}{|}^{2}\\ s.t. & \;C_{1}:\frac{\left(2^{\frac{p_{k}^{u}\left(L_{k}-L_{k}^{u}\right)}{B(E_{k}^{h}-E_{k}^{loc})}}-1\right)\delta_{bs}^{2}}{p_{k}^{u}}\leq |h_{k}^{u}\hat{\theta_{k}^{u}}{|}^{2},\\ \; & \;C_{9}:{\left[{\hat{\theta_{k}^{u}}}^{H}\hat{\theta_{k}^{u}}\right]}_{nm}=1,\forall n\in N. \end{array}$$

To address the nonlinear equality constraint problem in Equation (32), introduce variable $\hat{\Theta_{k}^{u}}={\hat{\theta_{k}^{u}}}^{H}\hat{\theta_{k}^{u}}$, $\hat{H_{k}^{u}}=h_{k}^{u}h_{k}^{u^{H}}$, transforming the problem into

(33) $$\begin{array}{cc} \; & \max_{\Theta_{k}^{u}}\;Tr\left(H_{k}^{u}\Theta_{k}^{u}\right)\\ s.t. & \;C_{1}:\frac{\left(2^{\frac{p_{k}^{u}\left(L_{k}-L_{k}^{u}\right)}{B(E_{k}^{h}-E_{k}^{loc})}}-1\right)\delta_{bs}^{2}}{p_{k}^{u}}\leq Tr\left(\hat{H_{k}^{u}}\hat{\Theta_{k}^{u}}\right),\\ \; & \;C_{9}:{\left[\hat{\Theta_{k}^{u}}\right]}_{nn}=1,\forall n\in N,\\ \; & \;C_{12}:\hat{\Theta_{k}^{u}}⪰0,\\ \; & \;C_{13}:Rank\left(\hat{\Theta_{k}^{u}}\right)=1. \end{array}$$

The solution approach is similar to above Equation (30).

## 5. Simulation Analysis

This section verifies through simulation the performance of the proposed scheme in IRS-assisted SWIPT-MEC system. The study considers a three dimensional (3D) coordinate deployment: BS at (10 m, 0, 0), IRS at (2 m, 6 m, 0), and IoT devices randomly placed within a circle centered at (0, 0) with a radius of 3 m. Additionally, all channels experience both large-scale and small-scale fading. The large-scale fading follows a path loss model given by

(34) $$L\left(d\right)=C_{0}{\left(\frac{d}{d_{0}}\right)}^{-\alpha },$$

where $C_{0}$ is the path loss at a reference distance of $d_{0}=l\;m$, and *d* is the actual link distance. In this study, the IRS to BS and IRS to IoT devices links are subject to Ricean fading with a Ricean factor of 5 dB, while the BS to IoT devices link follows Rayleigh distribution. The path loss exponents are assumed to be 2 for IRS to BS and IRS to IoT devices links, and 3.5 for BS to IoT devices links.Unless otherwise specified, the main simulation parameters are set as follows: number of IRS reflecting elements N=80, energy conversion efficiency $\eta_{k}=0.8$, effective capacitance coefficient $\kappa_{k}=10^{-28}$, noise power $\delta_{bs}^{2}=\delta_{k}^{2}=10^{-12}$ W, CPU cycles required per bit $C_{k}=500\;\mathrm{cycle}/\mathrm{bit}$, channel coherence time T=0.5s, maximum offloading transmit power $p_{max}^{u}=0.5\;W$, minimum offloading transmit power $p_{min}^{u}=0.1$ W, system bandwidth B=[320,400]KHz, and total computation workload L=[15,20]Kbits. Using these parameters, simulations are conducted using MATLAB (R2023b) software to evaluate the proposed scheme.

In simulation, three benchmark schemes are considered to compare with the proposed algorithm, validating its effectiveness:

1. Without IRS-assisted SWIPT-MEC: This system does not deploy IRS. The optimization of PS ratio, local computation workload, offloading transmit power of IoT devices, and CPU frequency are performed using the algorithm proposed in this paper.
2. IRS-assisted SWIPT-MEC with random phase shifts: The phases of IRS are randomly generated. The optimization of PS ratio, local computation workload, offloading transmit power of IoT devices, and CPU frequency are also conducted using the algorithm proposed in this paper.
3. IRS-assisted SWIPT-MEC with offloading only: The entire workload of the IoT devices is offloaded to the MEC server for computation. The IRS phase shifts, PS ratio, offloading transmit power of IoT devices, and CPU frequency are optimized using the algorithm proposed in this paper.

**Figure 4:**
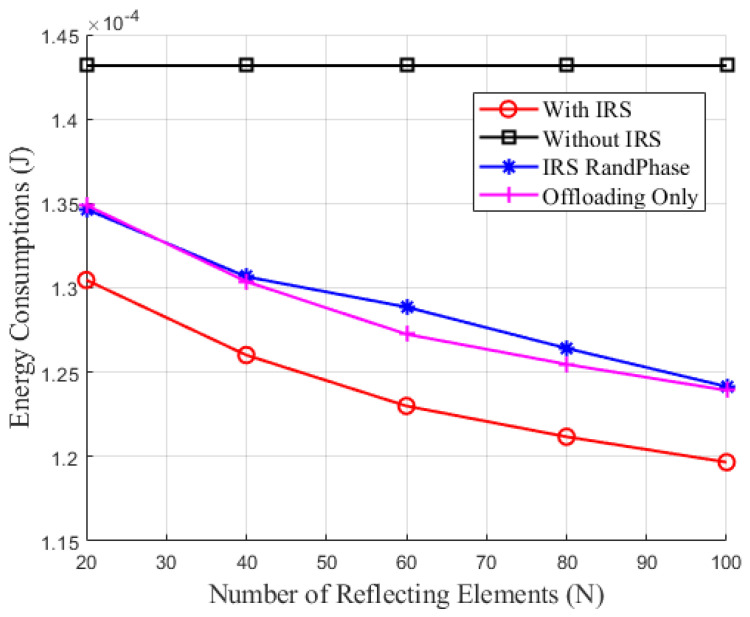
Energy consumption versus number of IRS reflecting elements.

Figure 4 illustrates the relationship between energy consumption and the number of IRS reflecting elements *N*. From Figure 4, it can be observed that, except for the scheme without IRS assistance, the energy consumption of IoT devices decreases as the number of IRS reflecting elements increases. This indicates that deploying an IRS can significantly reduce the energy consumption in SWIPT-MEC system for IoT devices. The reduction is due to the IRS enhancing signal quality and transmission efficiency in communication links by providing additional reflective paths. Specifically, as the number of IRS reflecting elements increases, the path diversity of the signal improves, allowing the transmission signal to reach the terminal more effectively. This results in reduced signal attenuation and multi-path interference, thereby lowering the transmit power requirements of the IoT devices. Further analysis reveals that the optimized IRS phase shifts scheme has stronger control capabilities compared to the IRS-assisted SWIPT-MEC with random phase shifts scheme. It can precisely adjust the path of the reflected signal, allowing it to form a more effective combination with the direct signal, thereby maximizing signal gain and link quality. This precise control not only enhances the reliability and stability of communication but also reduces the energy consumption of IoT devices. During the task offloading process, the energy required for fully offloading tasks under the optimized IRS phase shifts is lower than that under the random IRS phase shifts optimization scheme, but higher than that under the optimized IRS phase shifts joint offloading scheme. This is because the joint offloading scheme can flexibly allocate tasks, maximizing signal gain while ensuring computational efficiency, thereby minimizing overall energy consumption. These results comprehensively validate the effectiveness and superiority of the three proposed combined techniques in reducing energy consumption and improving system performance.

**Figure 5:**
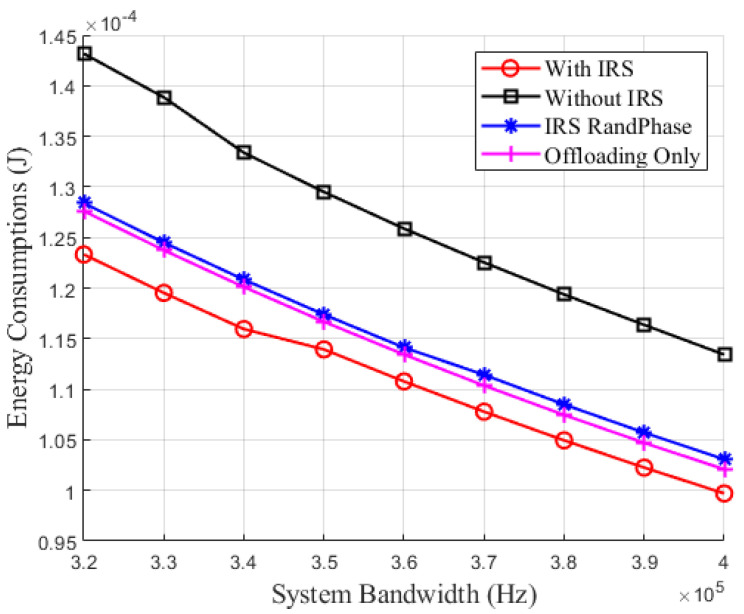
Energy consumption versus system bandwidth.

Figure 5 illustrates the relationship between energy consumption and system bandwidth. From Figure 5, it can be observed that as system bandwidth increases, the energy consumption of all four schemes shows a decreasing trend. This trend occurs because the increase in system bandwidth leads to a higher data offloading rate for IoT devices, allowing them to complete data transmission in a shorter time, thereby significantly reducing energy consumption during transmission. Additionally, the increase in bandwidth reduces the congestion in communication links, improving data transmission efficiency and further lowering energy consumption. Further analysis of the four schemes reveals that the optimized IRS phase shifts and partial task offloading schemes exhibit a clear advantage in reducing energy consumption. The reasons for this advantage are as follows: Firstly, the optimized IRS phase shifts scheme can precisely control the phase of the reflected signal, minimizing path loss and thereby enhancing signal gain and communication efficiency. Secondly, the partial task offloading strategy can flexibly adjust the amount and method of task offloading based on the available bandwidth. Compared to the other three schemes, the optimized IRS phase shifts and partial task offloading schemes more effectively utilize the benefits of system bandwidth and signal phase adjustment. As a result, these schemes can maintain lower energy consumption under different bandwidth conditions, demonstrating superior energy efficiency, especially in high-bandwidth environments, where the reduction in energy consumption is even more pronounced. These results fully validate the significant advantages and practical applicability of the optimization strategies in mitigating the impact of increased bandwidth on energy consumption.

**Figure 6:**
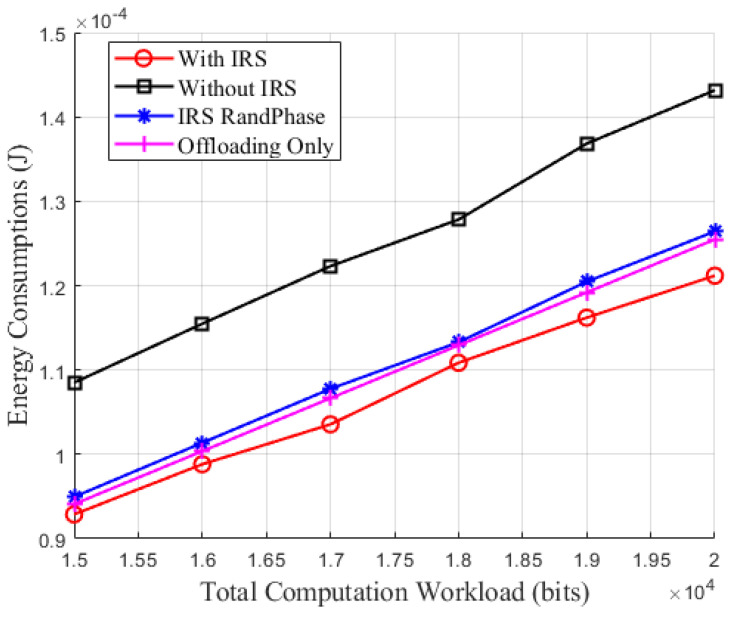
Energy consumption versus total computation workload.

Figure 6 illustrates the relationship between energy consumption and the total computation workload. As observed from the Figure 6, the energy consumption of all four schemes increases with the rise in total computational workload. This is because the increase in workload necessitates more computational resources from IoT devices, thereby escalating their energy consumption. Comparing the random phase shifts of the IRS with offloading only scheme assisted by the IRS, there is not a significant difference in energy consumption between the two. However, when comparing all four schemes, the proposed method in this paper demonstrates superior performance in the SWIPT-MEC system, showing consistently lower energy consumption as the total computational workload increases. This may be attributed to the optimization of the IRS phase shifts, which more effectively controls the signal propagation path, reduces signal attenuation and interference, thereby lowering energy consumption while ensuring efficient and reliable data transmission.

**Figure 7:**
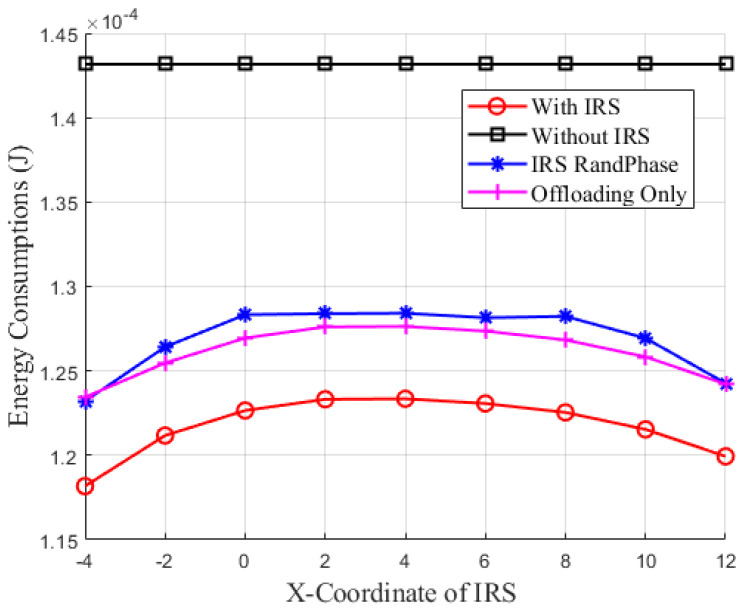
Energy consumption versus IRS position.

Figure 7 illustrates the relationship between energy consumption and IRS position. Specifically, it shows how the energy consumption of IoT devices varies with the IRS placed at different positions. The performance of the IRS improves as its *X*-coordinate increases, reaching a peak, and then starts to decline. In contrast, the SWIPT-MEC system without IRS assistance shows no change in energy consumption. This trend is due to the IRS achieving maximum reflection efficiency at its optimal deployment position, which significantly enhances system energy efficiency, thereby minimizing the energy consumption of IoT devices. When the IRS is positioned closer to the BS along the *X*-coordinate, there is a noticeable decrease in energy consumption, validating the optimal IRS deployment position for minimizing the energy consumption of IoT devices. Compared to other schemes, the proposed approach better leverages the reflective properties of the IRS, optimizing the system with the goal of minimizing IoT devices energy consumption.

## 6. Conclusions and Future Work

To extend the lifecycle of the IoT, this paper investigates the resource allocation problem in IRS-assisted SWIPT-MEC system. Considering constraints such as energy harvesting of IoT devices, PS ratio, CPU frequency, local computation workload, and IRS phase shifts constraints, we establish a multi-resource joint optimization model aimed at minimizing the energy consumption of IoT devices. The problem is decomposed into two sub-problems using an AO iterative algorithm based on block coordinate descent. Subsequently, an AO algorithm sequentially optimizes the variables in the sub-problems. First, we employ closed-form solutions to optimize the CPU frequency of IoT devices. Second, convex optimization tools, specifically CVX, are utilized to optimize the local computation workload of IoT devices. Third, we employ the Dinkelbach-based Lagrange dual method to optimize the optimal offloading transmit power of IoT devices. Furthermore, closed-form methods are used to optimize the PS ratio, and the SDR method is employed to optimize the uplink and downlink phase shifts of IRS. Simulation results demonstrate that compared to baseline solutions, the proposed approach can significantly reduce the energy consumption of IoT devices under different conditions.

The paper employed a FIFO approach for task computation in IoT devices and did not adequately consider priority-based task scheduling based on task urgency. It assumed that CSI was known, but accurately obtaining CSI in practical applications was often challenging, which might have led to discrepancies between theoretical analysis and actual performance. Future work could further investigate performance optimization issues such as IoT devices efficiency and computation rate within this system architecture. Considering the difficulty of obtaining perfect CSI, it would be valuable to evaluate and optimize system performance under conditions of unknown CSI to enhance the system’s practicality and robustness.

## Data Availability

Not applicable.
